# Community-based rehabilitation/community based inclusive development functioning during the COVID-19 pandemic: A secondary analysis of qualitative data

**DOI:** 10.1371/journal.pone.0296274

**Published:** 2024-01-05

**Authors:** Ansha Nega Ahmed, Reshma Parvin Nuri, Xiaolin Xu, Venkatesh Balakrishna, Alaa Sebeh, Carolyne Maholo, Heather Michelle Aldersey

**Affiliations:** 1 School of Public Health, Addis Ababa University, Addis Ababa, Ethiopia; 2 School of Rehabilitation Therapy, Queen’s University, Kingston, Ontario, Canada; 3 Community Based Rehabilitation (CBR) Global Network, Anantapur, Andhra Pradesh, India; 4 Disability Institution, United Nations Economic and Social Commission for Western Asia, Beirut, Lebanon; 5 Department of Community and Disability Studies, Kyambogo University, Kampala, Uganda; Florida Atlantic University Charles E Schmidt College of Medicine, UNITED STATES

## Abstract

**Introduction:**

The coronavirus (COVID-19) became a global pandemic in March 2020 and impacted nations worldwide not only because of the disease but also because the containment measures-imposed created ripple effects for the populations in each country. The COVID-19 pandemic disproportionately affected vulnerable groups, such as persons with disabilities. This study aimed to understand the impact of COVID-19 on the function of Community-Based Rehabilitation (CBR)/Community-Based Inclusive Development (CBID) across nations and for their target communities—persons with disabilities. The current article also described some measures CBR/CBID programs took in light of service closure to facilitate access to needed services for persons with disabilities.

**Methods:**

We conducted a secondary analysis of qualitative data to understand the impact of COVID-19 on the functioning of CBR/CBID programs and their target communities. The original qualitative data were collected through online dialogues among CBR/CBID partners across five regions of the world, facilitated for understanding of their practices on five other topics.

**Findings:**

COVID-19 significantly impacted the function of CBR/CBID programs across the world. Many services were halted due to public health measures, such as maintaining social distancing or lockdowns. The COVID-19 pandemic also had a negative impact on access to health, education and livelihood support for persons with disabilities. Additionally, many people with disabilities did not have access to COVID-19 related information and services like vaccines. However, we found that technology played a significant role in revitalizing CBR/CBID programs during COVID-19. CBR/CBID service providers across five regions used online platforms to disseminate information about COVID-19. Professionals also used technology to provide rehabilitation and educational services to people with disabilities through online platforms.

**Conclusion:**

Our findings suggest that technology can play a vital role in continuing many services (e.g., CBR/CBID) that cannot be offered in person during crises like COVID-19. However, it is important to remember that technology may not be accessible to many individuals with disabilities, specifically those who reside in rural areas and who experience adverse situations like financial constraints. Additionally, many persons with disabilities may not have the necessary knowledge and skills to use technology. CBR personnel must consider that before adopting technology to provide services under CBR programs.

## Introduction

In March 2020, the World Health Organization (WHO) declared that COVID-19 was a global pandemic [1]. Since then, the pandemic has presented us with exceptional challenges, and its impact will continue to be significant in the years to come [2]. Globally, almost all countries went into nationwide lockdown to manage the spread of COVID-19. The impact of COVID-19 was multidimensional and influenced society across various socioeconomic conditions [3, 4]. The pandemic and associated lockdowns were particularly challenging in the everyday lives of persons with disabilities due to the inequity in accessing necessary preventive and basic life supports, particularly in low-resource settings [4, 5].

Rehabilitation interventions are essential to prevent the onset of and complications from disability [6]. Rehabilitation is considered as high value as it aims to optimize functioning and reduce disability and this need is expected to rise in the future [7]. However, the types, distribution and accessibility of rehabilitation services vary by geographic, economic and social contexts. On top of this, as a response to the COVID-19 pandemic, various service providers, including those of rehabilitation services, were forced to think critically and shift their approach in various ways. For example, scholars [8, 9] summarised what happened when COVID-19 challenged the function of healthcare settings. This shift introduced an additional burden to the individuals, their families and service providers. Individuals with disabilities and their families encountered cumulative hardships even before the occurrence of the COVID-19, and the pandemic exacerbated them. For example, COVID-19 significantly impacted the function of community-level services that involve in-person engagement, including Community Based Rehabilitation (CBR) and Community Based Inclusive Development (CBID). In response to the pandemic, rehabilitation practitioners have developed innovative ways to deliver services, including developing guidelines specifically for physiotherapy service provision to inform rehabilitation practices within COVID-19 [10] and the use of robotic technology for home-based therapeutic services [11].

Community-Based Rehabilitation (CBR) is a strategy for community development that aims to enhance the lives of people with disabilities in the community. It is endorsed by the World Health Organization and others [12], and is a multi-sectoral approach that strives for the equalization of opportunities and social inclusion of people with disabilities in health, education, livelihood, and social sectors [12]. At the height of the pandemic and measures to reverse the spread of the virus, CBR/CBID programs, persons with disabilities, and families were also affected. There is so much to learn from the experiences of CBR/CBID programs during the pandemic and their roles in responding to the evolving nature of COVID-19. Studies in India explored and gave insight about the impact of the pandemic on service providers and vulnerable population, poor access to evaluation of needs, provision of services and monitoring and coordination of support stakeholders [13] and significant impact on their livelihoods [14]. However, little is documented about the experiences of programs that are primarily engaged within the community often through in-person engagement with persons with disabilities and their families, such as CBR/CBID. Therefore, it is important to explore how the pandemic impacted the CBR/CBID program activity and their target communities and how responsible and effective we were in responding to the unique experiences of persons with different disabilities in their communities. This article explored the experiences of CBR/CBID program implementers globally during the COVID-19 period. Specifically, in this study, we attempted to answer the following research questions: What was the impact of COVID-19 on the functioning of CBR/CBID programs? How did CBR/CBID programs respond to the challenges during COVID-19 era? And what can we learn from the strategic responses to inform future emergencies and other epidemics?

## Methods

This study is a secondary analysis of qualitative data that were originally collected via Zoom as part of global CBR dialogues from October to December 2020. Secondary analysis of qualitative data is an approach where researchers use an existing data set to answer a research question that is different from the questions that were asked in the original research [15]. We conducted the secondary analysis of qualitative data for the following reasons: (i) to maximize the use of existing data and (ii) to inform policy and practice related to CBR/CBID programs [16]. The section below provides a brief overview of how we collected the original qualitative data. Aldersey and colleagues (In Press) offer further information about the original study and methods.

### Overview of the original study/CBR dialogues

The primary purpose of the CBR dialogues was to facilitate learning from the experiences of CBR/CBID programs during the pandemic on five other thematic topics. The details of the CBR dialogues can be found elsewhere [17]. In brief, the dialogues were held in five different regions of the world managing CBR/CBID programs: Africa, the Americas, Asia, the Pacific, and the Arab regions. The dialogues were conducted in most convenient working language. There were options for simultaneous professional translation to some relevant language live captioning, including English. Also, local sign language interpretations were available, as required based on the profile of participants during the registration. Dialogues were focused on five different topics (i.e., community-level support; poverty, work and financial well-being; innovations in the community; justice, choice and power; and intersectionality and CBR/CBID). All five topics had specific guiding questions to facilitate the discussion. Table 1 provides an example of questions from one of the dialogue topics.

**Table 1:** Facilitated questions from innovation dialogues.

1. Please give examples of innovation from your CBR Program of how it promotes an inclusive innovation culture/practice in complying with CRPD and promoting SDGs in: a) personal assistance; b) rehabilitation services at home and in the community, including assistive devices; c) inclusive play for children; d) inclusive education; e) inclusive leisure and recreational activities and sports; f) livelihoods/employment/entrepreneurship; g) communication; h) advocacy; i) political participation; j) social mobilization. 2. How could the good practice in your CBR/CBID Program be rolled out more widely? 3. Please list types of innovation, e.g., using technology, local material, and storytelling. 4. What are the barriers and drivers of innovation in CBR/CBID in communities? How can we remove the barriers and increase the impact of the drivers? 5. How can we ensure that everyone has equal access to innovations? 6. In the last two years, what changes have you seen in collaboration across sectors (such as health, education, and employment), levels of government (local, regional and national) and involving a broad range of stakeholders, including persons with disabilities in their organizations? 7. Please share to what extent persons with disabilities and their organizations actively participate in your work and what difference their contribution has made to the quality of your CBR program.

The research protocol for conducting the CBR dialogues was reviewed, and ethical clearance was obtained from the Queen’s University Health Sciences and Affiliated Teaching Hospitals Research Ethics Board (HSREB). Informed verbal consent was obtained prior to each dialogue session with a statement, read by the chairperson of the dialogue, that describe “participants’ responses would be documented and recorded and would factor into an overall analysis and future publications and presentations for global communication.” Participants were asked to reflect if their response is “yes” when they agreed or “no” when they did not wish their responses to be used in the analysis. At the time of the dialogue, we received “yes” responses from all. In addition, we gave participants an option to inform us via email to either of the senior authors (AN and/or HA) if they had any reservation in having their responses be used in the analysis for future publication and presentations. However, we didn’t receive any opposing statement from any participant, either during or after the dialogues.

At the beginning of each dialogue, participants were informed that the session would be recorded, and data would be used for report preparation and future possible publications or presentations. After each dialogue, authors (i) prepared transcriptions, (ii) translated them into English (for languages other than English) and (iii) removed identifiable names from the transcript.

### Secondary data analysis process for this study

We imported transcripts into NVivo12—qualitative data management software—for data analysis. We employed content analysis with an inductive approach—a ground-up approach that allows researchers to develop themes from the raw data [18] to answer the new research questions, related to COVID-19. In content analysis, the research team was engaged in the following activities: (i) reading the transcripts, (ii) identifying meaningful units from data, (iii) labelling meaningful units by formulating codes, (iv) grouping codes into categories according to their similarities and differences, and (v) grouping categories into themes based on trends and patterns [19, 20].

### Findings

These findings are presented based on all the 25 dialogues across the five different regions, five two-hour dialogues on five discussion topics. We pulled important threads that are related to CBR/CBID services and COVID-19 pandemic from all the 985 participants (458F, 492M and 35 Other/Not Specified) in all the five topics.

During dialogues, participants described the impact of COVID-19 on the function of CBR/CBID programs which are organized into three themes: (i) impact of COVID-19 on the function of the CBR/CBID programs; (ii) impact of COVID-19 on the lives of people with disabilities; and (ii) strategies used to revitalize CBR/CBID programs.

### Impact of COVID-19 on the function of the CBR/CBID programs

Participants shared that COVID-19 significantly impacted their CBR/CBID programs and the way they functioned. Participants specifically noted that the public health measures (e.g., complete lockdown or maintaining social distance) that were introduced to restrain the spread of the coronavirus disrupted CBR/CBID activities related to health, education and livelihood for people with disabilities. In most places, the CBR/CBID activities were halted despite the need for in-person services for people with disabilities. The physical restrictions/lockdown also challenged CBR/CBID activities related to stakeholders’ meetings and collaborations. A participant from Asia region recounted:

*The last two years have been difficult … most of the development work had come to a halt because we had to deal with COVID…*. *centers are closed*. *(Asia Region*)

Participants also cited financial constraints as a major challenge to keep CBR/CBID programs running during the pandemic. They stressed the need for financial support not only to run their programs but also to address the needs of persons with disabilities and their families. However, some participants noted that some CBR/CBID programs received direct and indirect financial support from funders or other organizations, but this was reflected with a concern about the sustainability of these supports. A participant from Arab Region noted: *we received some financial aid from organizations and not*, *of course*, *from the government*. *Indeed*, *these worries [us] because there is no sustainability in this kind of [supports]*. In some CBR programs, service providers employed different strategies to raise funds in order to keep their programs running. CBR/CBID programs in Pacific Region and Africa Region were able to secure grants from different sources to address the financial needs of people with disabilities. Also, a participant from the Americas Region described how they generated funds by selling in-kind donations they received from their members:

*Some of the members donated their bags and supplies of new things and we sold them*, *and we auctioned them*. *And it’s mostly about fundraising…because of the membership of the charity*, *we connect with very prominent family members*. *(Americas Region*)

### Impact of COVID-19 on persons with disabilities

Almost all participants cited that COVID-19 significantly impacted the lives of people with disabilities, including children. They specifically noted that persons with disabilities and their families were exposed to stressful situations in terms of health, livelihood and education during the pandemic. Many people with disabilities did not have access to information, preventative measures (e.g., vaccines) and essential services related to COVID-19. A participant from African Region recounted: *during COVID*, *persons with disabilities experienced a lot of discrimination*.*… many people with disabilities were kept behind without any information on how they can have access to COVID-related measures*. Consequently, some people with disabilities died from COVID-19. A participant from the Americas Region described this painful situation: *some persons with disabilities died because of discrimination and lack of access to health with the emergency of the COVID pandemic*. Some participants believed that persons with disabilities’ limited access to COVID-19 related information and services resulted from discriminatory attitudes toward disability or lack of inclusiveness. Participants further stated that these negative attitudes already existed in society; the pandemic just aggravated them and left persons with disabilities in dire situations. A participant from Pacific Region summed up this issue: *COVID-19 has exposed the world to what persons with disabilities already commonly experience in their everyday lives*. *That is poverty*, *isolation*, *mobility restrictions*, *and lack of access to basic services such as health*, *food*, *education*, *employment and other aspects of community life*. *This has made us more vulnerable*.

In addition to the experiences of discrimination and limited access to health services, the pandemic significantly affected the livelihoods of people with disabilities. Participants reported that many people with disabilities lost their jobs due to the lockdown, especially in the tourism sector. This situation, along with limited or no support from the government, left people with disabilities in a dire financial situation and pushed them further into poverty. The lockdown had a catastrophic effect on people with disabilities who could not afford to stay out of economic activities for basic income. A participant from Pacific Region explained: *When COVID-19 hit*, *what it did*, *[since] the Cook Islands depends on the tourism industry and we suffered a lot and there was no buffer…No job–no employment*, *no social protection schemes*.

Participants also narrated that the lockdown and restriction on in-person communications seriously affected children with disabilities’ access to education. The effects persisted for children with disabilities even after the education sector made some flexibility to offer education virtually. Participants noticed that virtual education was challenging for students with visual impairment because of the unique challenges experienced by these individuals. A participant from Arab Region explained why virtual education was challenging for children with visual impairment: *it is known that the schools closed*, *and blind students studied using Braille … Braille depends on touch*, *meaning online education won’t work*.

### Strategies used to revitalize CBR/CBID programs

In the dialogues, participants discussed how crises like COVID-19 taught them to find innovative solutions rather than relying on routine approaches. Participants specifically noted that the crisis moment enabled them to adopt new initiatives and employ creative approaches in providing CBR/CBID services to address challenges experienced by persons with disabilities and their families during the pandemic. A participant from the Arab Region recounted: *it is the pandemic that taught us lessons and gave us many ideas to create new things… [COVID-19] gave us opportunities to create new patterns in community work that we were not used to do before*. *So*, *congratulations on the ’side effects’ of COVID-19*. In terms of new initiatives, participants mostly discussed that many CBR/CBID programs took measures to address the immediate needs of persons with disabilities (e.g., ensuring access to vaccines, medicine and food). For instance, a participant from Arab Region highlighted how they liaised with the public sector and civil society to facilitate persons with disabilities’ access to vaccines: *[In] Jordan*, *[a CBR program] cooperated with the Ministry of Health … there was an agreement between the Council and the Ministry of Health to vaccinate people with disabilities who are unable to reach health centers*. *They reached an agreement that persons with disabilities would be vaccinated…*. *The health team used to reach their locations via mobile*. In some places, CBR/CBID programs themselves distributed medicine and food free of cost.

*[We] provide medicines for free*, *for short periods…through the associations*, *to providing food and also financial support through the nearest port they could reach during the pandemic*. *(Arab Region*)

Besides new initiatives, participants noted that many CBR/CBID programs revitalized their regular services and used existing systems to reconnect with persons with disabilities and their families. Participants also highlighted that arranging financial aid, exploring employment options, and creating entrepreneurship opportunities for persons with disabilities, including remote work arrangements, were key areas of their efforts to revitalize the CBR/CBID programs in the early stage of the pandemic. Additionally, participants mentioned that increased awareness, advocacy and capacity building were other areas they strengthened when they attempted to revitalize their services. Participants noted that they emphasized advocacy work to restore the destructed spirit of inclusiveness and to enhance necessary support for persons with disabilities.

Participants cited that technology played a significant role in revitalizing CBR/CBID programs and connecting with people with disabilities. Specifically, participants reported using Zoom, WhatsApp, Messenger, and social media to educate people with disabilities on how to access vaccines and other services. Some participants also reported that their routine rehabilitation services were resumed using technology. Specifically, participants noted that some CBR programs across Africa, Asia and Arab regions were able to provide health-related services, such as telephone counselling and online exercise programs for individuals with disabilities. A participant from the Asia Region stated: *During COVID… [we] have started an online exercise program for children with autism*, *psychosocial disabilities and intellectual disabilities*. *And in my personal opinion*, *the significance was that they didn’t just do it for their own community*, *but it was with ten different countries*. Another participant from Africa Region noted: *we also saw from Ethiopia*, *where resilience for the mentally challenged*, *there was a lot of support*, *especially during COVID-19*. *They changed the approach from the traditional*, *you know*, *to one-to-one telephone counselling*. *Volunteers worked on a shift basis so that services could continue during COVID-19*.

Some participants indicated that CBR/CBID programs in some places used technology to facilitate education for children with disabilities. A testimony from the Arab region showcases an innovative approach to address challenges experienced by students with visual impairment to attend their study virtually: *we hosted them in the Supreme Council…we took into account the safety measurements and allowed the teacher to teach them… blind students did not stop learning … and we celebrated their graduation*. A participant from the Arab Region also summarized their initiatives to facilitate education for children with disabilities: *It was about raising awareness for the families… we decided to rely on social media through creating a group on WhatsApp*, *on Facebook and creating official websites… parallel initiatives to make distance education fair for students with visual disabilities and deafness*, *by creating audio curricula*, *and sign language curricula… simplifying the curricula for people with intellectual disabilities… I mean*, *there were beautiful initiatives*, *recording videos and sending them to families… to continue teaching skills to children so that they won’t stop receiving education*. However, participants discussed access to technology, or digital literacy, was a challenge for many students with disabilities, especially among rural residents. A participant from Africa region described this challenge: *the communication systems were very poor in many rural areas*. *Very often there is no power*. *There is very poor internet connection*. *Sometimes there is very poor cell phone connection*. *And that makes communication very difficult*, *particularly in those rural areas*.

One of the interesting aspects discussed in the dialogues was related to data utilization. Participants shared that in many places, CBR/CBID leaders used research findings, including personal stories, about the impact of COVID-19 on persons with disabilities to strengthen their advocacy work. Participants also noted that such strategies helped secure more resources for persons with disabilities during the pandemic. For instance, a participant from the Americas shared how they successfully drew the government’s attention to people with disabilities when they used data in their advocacy work: *in Antigua*, *by sharing data with the government about COVID software…they now are looking to map the disabled person in Antigua*.

## Discussion

The findings of this study are consistent with international research that highlighted the impact of COVID-19 on service provision. Similar to other domains of services, COVID-19 had devastating impact on CBR/CBID programs and their target populations—persons with disabilities. Specifically, physical restrictions due to lockdowns posed serious challenges to personal assistance and in-person rehabilitation services for persons with disabilities. In many countries, rehabilitation services were completely stopped or provided in a reduced capacity due to budget cuts or not seen as essential services in the course of the pandemic, which had a detrimental effect on persons with disabilities (e.g. increased morbidity or mental health issues) [8, 21]. In line with previous literature [21, 22], the present study highlights a need to enhance access to rehabilitation services for persons with disabilities and ensure adequate financial resources for rehabilitation services in future crisis management like COVID-19. Besides rehabilitation services, it is also important for CBR/CBID programs to facilitate persons with disabilities’ access to other services (e.g., livelihood and education), and promote community participation following safety guidelines [23].

Like what has been reported in other studies [24, 25], we also found that people with disabilities experienced numerous barriers in accessing health, education and livelihood services during the pandemic. The United Nations [26] reported that many persons with disabilities could not receive the formal or informal support they needed due to lockdowns, which largely impacted their autonomy, health, and lives. Similarly, the dialogues also revealed that persons with disabilities did not have access to essential services like COVID-19 related information, which clearly indicates a lack of inclusiveness in service provision and affirms pre-existing inequalities and discrimination against disability. Therefore, it is recommended that any disaster management efforts, like COVID-19, are disability-inclusive and information is available in accessible formats (e.g., ensure sign language interpreter and service provider wear transparent mask to allow lip reading). It is also recommended to provide training to service providers about the diverse needs of people with disabilities to safeguard them against discrimination and prevent inequities in service provision [27].

While COVID-19, specifically the lockdown, had a widespread impact on most people with disabilities, the restriction on human movement disproportionately affected people with disabilities who had to rely on others for activities of daily living [28]. In particular, persons with disabilities who had visual impairments experienced difficulties accessing required medication, food, or assistance in activities of daily living, such as bathing, dressing, feeding, and toileting, when they were required to quarantine or when their caregivers/ personal assistants were quarantined or fell ill [29]. Therefore, it is essential to remember that any measures to restrain the spread of diseases similar to COVID-19 should not prompt segregation; instead, safety measures need to introduced so that these individuals can interact with their caregivers for essential services. Additionally, attention should be paid to the well-being of these individuals in times of crisis and measures need to be in place to enable essential activities [30].

We found that technology played a significant role in increasing persons with disabilities’ access to disability support services. Service providers used professional media to raise awareness about COVID-19. In many places, service providers were able to provide rehabilitation services (e.g., home exercise for children with disabilities) via Zoom. The dialogues also indicated that the education of children and youth with disabilities benefited from the utilization of digital technologies. The potential of using digital technologies, such as videos, mobile messaging, social media, and eLearning, to improve the accessibility and sustainability of CBR/CBID has been widely recognized in the literature [22, 31, 32]. Specifically, robotics rehabilitation [11] and telerehabilitation [33, 34] were helpful in facilitating rehabilitation services during COVID-19. Therefore, future CBR/CBID programming might consider including technology access and literacy as critical components in service planning. However, some concerns about telerehabilitation or tele-education include the lack of internet access—fast internet and persons with disabilities’ ability and safety to undertake telerehabilitation or tele-education [35]. This is particularly challenging for people with disabilities who reside in remote or resource-poor communities and are unable to navigate technologies because of their disability or low literacy. Therefore, it is essential to recognize and accommodate the diverse needs of people with disabilities while using technology to provide services or education to these individuals. For instance, persons with communication difficulties may need unique solutions for telerehabilitation or tele-education to be effective. Similarly, the virtual interfaces need to be customized to accommodate the needs of persons with mental illness or autism spectrum disorders so that these individuals are not left behind in digital world [36].

The study also highlights the importance of collaboration among stakeholders to manage challenges arising from the pandemic. According to Sivakumar and colleagues [37], the collaboration that a rural CBR program in India developed over the years with various stakeholders helped to ensure the continuity of care for persons with mental illness during COVID-19. In addition, the collaboration between the government and private partners can promote Internet access and cyber security for telehealth [8]. However, it is sometimes challenging to collaborate within and across agencies during health crisis. Regular communication and discussion about shared values with relevant stakeholders are vital to facilitating collaboration across agencies.

### Limitations

This research has a number of limitations. Firstly, we used secondary data to understand the impact of COVID-19 on the function of CBR/CBID programs, and on persons with disabilities and families. It is possible that we missed some nuances regarding the impact of COVID-19 on CBR/CBID programs and on the lives of people with disabilities due to the nature of the study. Despite these challenges, the secondary data analysis allowed us to understand the impact of COVID-19 on the CBR/CBID program and its beneficiaries which will inform policy and practice decision-making. In addition, the virtual data collection could have a limitation to further explore a specific matter.

## Conclusion

This study is one of the first to explore the impact of COVID-19 on CBR/CBID programs and persons with disabilities, with global perspectives. We found that COVID-19 greatly affected CBR/CBID programs and persons with disabilities’ access to CBR/CBID services. The restrictions which followed to mitigate the transmission of the disease came with many challenges for persons with disabilities and their families as well as the organizations of and for persons with disabilities among other service providers. The provision of digital platforms or technology devices showed promising results in delivering telehealth care and education during the pandemic without placing them at increased risk of contracting COVID-19. Customized solutions to unique patient needs are required to improve healthcare access and outcomes for persons with disabilities.
